# Investigating the response mechanisms of bread wheat mutants to salt stress

**DOI:** 10.1038/s41598-023-45009-2

**Published:** 2023-10-30

**Authors:** Hedayat Karimzadeh, Azam Borzouei, Behnam Naserian, Seyyed Ali Tabatabaee, Mohammad Reza Rahemi

**Affiliations:** 1 Agriculture Research School, Nuclear Science and Technology Research Institute, P. O. Box: 31485-498, Karaj, Iran; 2 Seed and Plant Improvement Research Department, Yazd Agricultural and Natural Research and Education Center, AREEO, Postal Box: 89165-571, Yazd, Iran

**Keywords:** Plant breeding, Plant physiology, Plant stress responses

## Abstract

Mutation breeding is among the most critical approaches to promoting genetic diversity when genetic diversity is narrowed for a long time using traditional breeding methods. In the current study, 15 wheat mutants created by gamma radiation and three salt-tolerant wheat cultivars were studied under no salinity stress (Karaj) and salinity stress (Yazd) during three consecutive growing seasons from 2017 to 2020 (M05 to M07 generations mutants). Results showed that salinity induced lipid peroxidation and enhanced ion leakage in all genotypes however, M6 and M15 showed the least ion leakage increment. It was also observed that the activity of antioxidant enzymes including SOD, CAT, POX, APX and GR increased with salinity; the maximum increase in antioxidant activity was belonged to M15, M09, M06 and M05. All genotypes had higher protein content in salinity stress conditions; M07 and M12 showed the lowest (1.8%) and the highest (17.3%) protein increase, respectively. Zeleny sedimentation volume increased under salinity stress conditions in all genotypes except M06, C2, C3, and M07. The result indicated that salinity stress increased wet gluten in all genotypes. M10 and M08 showed the highest (47.8%) and the lowest (4%) wet gluten increment, respectively. M06 and M11 mutants showed the lowest (6.1%) and the highest (60.7%) decrement of grain yield due to salinity stress, respectively. Finally, M04, M05, M07, M13, and M14 were known as genotypes with high grain yield in both no salinity and salinity stress conditions. In other word, these genotypes have higher yield stability. The results of the current study revealed that gamma irradiation could effectively be used to induce salinity tolerance in wheat.

## Introduction

Wheat (*Triticum aestivum* L.), maybe the most important crop worldwide, is widely cultivated in most parts of the world. Due to its accounting for 30% of world grain production and 45% of cereal nutrition, wheat is an critical crop in human nutrition^1^. Worldwide cultivation area, production quantity, and grain yield of wheat are 221 million hectares, 770 million tons, and 3.5 tons per hectare, respectively^2^. In recent years; the wheat yield has increased by a slope of 0.5% per year, which is much less than the 1.4% expected to be sufficient to feed a growing human population^3^. There are various limitations in wheat production that prevent wheat to reach its potential grain yield. In addition to water and nutrient deficiencies, soil salinity is one of the most essential factors in reducing wheat grain yield worldwide^4^. Wheat is also the most important agricultural crop in Iran and with a cultivated area of 6.9 Mha (2.4 Mha irrigated and 4.5 Mha rainfed), it has the largest cultivated area among agricultural crops^5^. On the other hand, most of the area of Iran (more than 88%) has an arid and semi-arid climate^6^, and the occurrence of salinity stress and its annual intensification is inevitable in these areas, therefore, in most parts of Iran, wheat faces salinity stress (various degrees from low to very high).

Electrical conductivity of saturated paste extract above four dS m^−1^ (about 40mM NaCl) is considered a criterion for soil salinity. About 19.5% of irrigated lands (about 45 million ha) and 2.1% of rain-fed areas (about 32 million ha) are faced with different levels of salinity stress. Indeed, it is estimated that more than 50% of the world's arable land will be saline by 2050^7^. It was reported that grain yield of durum wheat decreased due to salinity stress by up to 50% in dray land^8^, while reduction of bread wheat grain yield due to salinity stress is estimated up to 88% in irrigated lands. Initially; seed germination would be significantly decreased by salinity stress, crop growth and development would be adversely affected then and would lead to grain yield reduction. In addition, salinity stress disturbs photosynthesis, cell membrane activity, hormonal balance, water, and nutrient uptake, and enzymatic activities^9^. Salinity stress not only reduces grain yield but also changes the bakery quality of wheat grain. It was reported that salinity stress increases wet and dry gluten content and grain protein of wheat^10^. A study of salt-tolerant and salt-sensitive wheat genotypes revealed that salinity slightly increased the grain quality of salt-tolerant while it did not affect the grain quality of salt-sensitive genotypes^11^.

By inducing the effects of drought stress as well as oxidative stress, salinity causes damage to membranes, DNA, proteins and lipids^12^ and disrupts vital processes such as photosynthesis and respiration^13^. Under salinity stress conditions the balance between the production and scavenging of reactive oxygen species (ROS) is disrupted and the plant experiences oxidative stress^14,15^. Therefore, the plant develops antioxidant systems (enzymatic and non-enzymatic) to deal with the increase in the amount of ROS^16^. It also has been well established that a more effective antioxidant system lead to a greater tolerance to salinity stress^17,18^.

In addition to the severity and timing of salinity stress occurrence, plant type is also an important factor in responding to salinity stress. Different crops or different varieties of crop respond to salinity stress differently^19^. Plant breeders are trying to select more stress-tolerant genotypes in different environmental conditions. The prerequisite for plant breeding is high species diversity and diverse germplasm. When genetic diversity is narrowed for a long time using traditional breeding methods, induced mutations (mutation breeding) are among the most critical approaches to promoting genetic diversity^20^. There are more than 2300 mutant varieties of all plant species that are officially released and recorded in the Food and Agricultural Organization/International Atomic Energy Agency (FAO/IAEA) Mutant Varieties Database. The development of radiation mutations involves using X-rays, beta rays, gamma rays, ion rays, lasers, neutrons, and electron beams. Meanwhile, gamma rays were widely used to induce mutation in crops and resulted in the release of about 50% of the above-mentioned crop varieties^21^. Source and dose of application are critical factors in induction genetic diversity using mutagenic agents^22^. The procedure includes using different doses of gamma rays to create mutants; selecting the superior mutants based on morphological, physiological, and yield during successive generations. Finally, the more compatible mutant with the studied environmental conditions will be selected for cultivation in that area.

In recent years, the increase in temperature and decrease in rainfall has led to an increase in evapotranspiration, and as a result, an increase in salinity, thus the area of agricultural lands under salinity stress is increasing every year. The current varieties are not able to maintain the production potential under increasing salinity conditions, so it is necessary to identify and release cultivars that are more tolerant to salinity stress. In the present study, grain yield, biochemical traits, and grain quality of different bread wheat mutants (created by gamma irradiation) were evaluated under salinity stress and no salinity stress conditions to find most suitable mutant(s) for salinity stress conditions.

## Results

### Biochemical traits

Our finding showed that hydrogen peroxide (H_2_O_2_) content was higher in salinity stress in all studied genotypes (Table 1). Results indicated that C1 and M15 had the lowest H_2_O_2_ increment (80% on average) due to salinity stress while M10 and M11 had the highest increase (1.2 times on average) of H_2_O_2_ content. In addition, our results showed that salinity stress enhanced MDA content in all genotypes (Table 1). M11 showed the most enhancement of MDA (more than two times) whereas increment of MDA content was the lowest in C1, M15, M06, and M02 (1.25 times on average). Ion leakage was increased in all genotypes due to salinity stress (Table 1). It was observed that C1, M06, and M15 had the lowest ion leakage (73% on average) while M11 showed the highest ion leakage (1.4 times).

**Table 1:** H_2_O_2_, MDA, and Ion leakage of studied wheat genotypes (means and analysis of variance) in Karaj (no salinity stress) and Yazd (salinity stress).

	H_2_O_2_ ± SE (nmol mg^−1^ protein)		MDA ± SE (nmol g^−1^ fw)		Ion leakage ± SE (%)	
	Karaj	Yazd	Karaj	Yazd	Karaj	Yazd
C1	3.42 ± 0.07 a	6.11 ± 0.13 a–c	4.85 ± 0.15 a	10.97 ± 0.16 ab	32.4 ± 1.40 a	54.7 ± 0.56 ab
C2	2.82 ± 0.15 b	6.01 ± 0.13 b–e	3.54 ± 0.13 b	10.53 ± 0.28 a–d	22.7 ± 1.86 b	51.8 ± 1.39 a–d
C3	3.08 ± 0.06 ab	6.52 ± 0.12 a	3.95 ± 0.09 b	11.12 ± 0.44 a	27.1 ± 1.14 ab	57.4 ± 2.39 a
M01	2.86 ± 0.09 b	6.10 ± 0.13 a–c	4.13 ± 0.16 ab	10.58 ± 0.14 a–c	23.3 ± 1.07 b	53.8 ± 1.07 a–c
M02	3.12 ± 0.13 ab	5.84 ± 0.15 c–f	4.08 ± 0.19 ab	9.19 ± 0.25 h	24.7 ± 1.66 b	47.2 ± 1.25 d–g
M03	3.00 ± 0.07 ab	5.76 ± 0.12 c–f	4.03 ± 0.15 b	10.01 ± 0.26 c–g	26.8 ± 1.23 ab	50.4 ± 1.81 b–f
M04	2.96 ± 0.05 ab	5.91 ± 0.22 c–f	4.06 ± 0.13 b	9.70 ± 0.19 e–h	25.2 ± 1.31 b	48.1 ± 1.47 c–g
M05	2.99 ± 0.09 ab	5.62 ± 0.13 d–f	3.90 ± 0.09 b	9.44 ± 0.21 gh	25.6 ± 1.14 b	47.4 ± 2.28 d–g
M06	2.99 ± 0.07 ab	5.93 ± 0.20 c–f	4.07 ± 0.13 ab	9.20 ± 0.25 h	26.3 ± 1.41 b	44.5 ± 1.72 fg
M07	2.84 ± 0.09 b	5.91 ± 0.19 c–f	3.87 ± 0.15 b	9.48 ± 0.24 f–h	22.7 ± 1.01 b	45.2 ± 1.64 e–g
M08	3.03 ± 0.12 ab	6.45 ± 0.13 ab	4.17 ± 0.19 ab	11.15 ± 0.25 a	26.8 ± 1.53 ab	56.2 ± 1.50 ab
M09	2.98 ± 0.10 ab	5.55 ± 0.19 f	3.78 ± 0.10 b	9.54 ± 0.35 f–h	24.5 ± 0.78 b	46.8 ± 2.90 d–g
M10	2.78 ± 0.11 b	6.06 ± 0.13 b–d	3.92 ± 0.19 b	10.24 ± 0.28 b–f	23.6 ± 1.48 b	50.7 ± 2.19 b–e
M11	2.79 ± 0.13 b	6.21 ± 0.13 a–c	3.57 ± 0.24 b	11.12 ± 0.24 a	22.1 ± 1.56 b	54.0 ± 1.65 a–c
M12	3.11 ± 0.11 ab	6.10 ± 0.15 a–c	4.17 ± 0.16 ab	10.38 ± 0.17 a–e	25.4 ± 2.11 b	51.0 ± 1.50 b–e
M13	2.93 ± 0.08 b	5.76 ± 0.14 c–f	3.94 ± 0.13 b	9.78 ± 0.35 d–h	25.6 ± 1.01 b	47.6 ± 2.00 d–g
M14	2.94 ± 0.11 b	5.59 ± 0.08 ef	3.96 ± 0.18 b	9.51 ± 0.27 f–h	24.0 ± 1.62 b	45.8 ± 1.72 e–g
M15	3.06 ± 0.06 ab	5.53 ± 0.19 f	4.11 ± 0.16 ab	9.27 ± 0.36 gh	24.3 ± 1.42 b	43.8 ± 2.90 g
Region	709.5***		2975.5***		49,114.0***	
Year	0.013 ns		0.1 ns		0.8 ns	
Region*year	0.042 ns		0.1 ns		6.0 ns	
Genotype	0.5**		2.8***		129.0 ***	
Region*genotype	0.5*		2.4***		75.0 *	
Year*genotype	0.2 ns		0.4 ns		21.5 ns	
Region*year*genotype	0.2 ns		0.5 ns		29.9 ns	
CV	8.4		9.1		13.0	
χ^2§^	0.05 ns		0.02 ns		0.2 ns	

For each trait, in each region, means with the same letter are not significantly different (slicing method, p < 0.05).

*ns* not significant.

*, **, and ***; significant at0.05, 0.01, and 0.001 probability level, respectively; §; Bartlett’s test for homogeneity.

Enzymatic antioxidant activity was increase by salinity stress in all studied genotypes (Table 2). Results showed that the highest SOD activity increases due to salinity stress was belonged to C1, M05, M09, and M16 (78% on average) whereas SOD activity increment by salinity stress was the least in M11 and C2 (49% on average). Increasing of CAT activity due to salinity stress was higher in comparison to SOD activity so that CAT activity increment was 1.2 times on average (average of all genotypes) while increasing of SOD due to salinity was 66% on average. Our results showed that M02 and M06 had the highest (1.5 times) and M11 had the lowest (88%) CAT activity increment due to salinity stress. POX activity showed the most increment due to salinity stress among all measured enzymatic antioxidants (Table 2). C1 (4.5 times) and M11 (2.5 times) showed the most and the least POX activity enhancement due to salinity stress, respectively. Our results also showed that similar to POX activity, M11 had the least increment of APX (70%) and GR (52%) activities due to salinity stress whereas C1 had the most increases of APX (1.2 times) and GR (97%) activities.

**Table 2:** SOD, CAT, POX, APX, and GR of studied wheat genotypes (means and analysis of variance) in Karaj (no salinity stress) and Yazd (salinity stress).

Genotype	SOD ± SE (u mg^−1^ protein)		CAT ± SE (u mg^−1^ protein)		POX ± SE (u mg^−1^ protein)		APX ± SE (u mg^−1^ protein)		GR ± SE (u mg^−1^ protein)	
	Karaj	Yazd	Karaj	Yazd	Karaj	Yazd	Karaj	Yazd	Karaj	Yazd
C1	135.1 ± 3.1 c	240.1 ± 3.7 c–e	69.6 ± 1.4 b	161.6 ± 1.9 fg	13.6 ± 0.9 b	75.8 ± 1.1 f–h	84.1 ± 2.2 c	185.2 ± 3.3 d–f	168.0 ± 3.5 c	331.2 ± 5.8 f–i
C2	161.0 ± 3.8 a	240.7 ± 6.7 c–e	84.9 ± 2.7 a	171.7 ± 5.1 d–f	22.4 ± 1.6 a	77.9 ± 1.8 e–g	106.0 ± 4.0 ab	194.1 ± 7.4 b–f	204.9 ± 9.4 ab	339.2 ± 4.6 e–i
C3	148.3 ± 2.3 a–c	230.0 ± 4.4 e	78.7 ± 1.6 ab	158.2 ± 4.1 g	19.3 ± 1.1 ab	71.5 ± 2.8 h	97.0 ± 2.5 a–c	179.8 ± 6.2 f	201.7 ± 4.6 ab	315.4 ± 8.1 i
M01	149.8 ± 3.3 a–c	240.7 ± 3.7 c–e	82.2 ± 2.2 ab	168.7 ± 4.5 e–g	20.3 ± 1.4 a	76.6 ± 1.3 f–h	103.3 ± 2.9 ab	192.3 ± 5.7 c–f	209.3 ± 5.1 ab	343.9 ± 6.5 d–h
M02	145.1 ± 3.5 bc	249.5 ± 4.0 b–d	78.4 ± 2.8 ab	191.5 ± 3.5 ab	19.6 ± 1.5 ab	81.9 ± 2.2 b–f	100.9 ± 3.4 a–c	191.9 ± 3.7 c–f	197.3 ± 7.9 ab	373.1 ± 6.9 ab
M03	152.6 ± 3.9 ab	251.9 ± 8.8 a–d	77.1 ± 2.2 ab	180.8 ± 6.9 b–e	19.7 ± 0.9 ab	78.2 ± 1.4 d–g	98.2 ± 3.3 a–c	193.7 ± 6.4 b–f	193.8 ± 5.3 a–c	353.5 ± 11.1 d–g
M04	149.8 ± 2.2 a–c	254.1 ± 5.7 a–c	78.9 ± 1.3 ab	183.8 ± 4.6 a–d	20.5 ± 1.1 a	84.1 ± 1.4 a–d	96.4 ± 3.1 a–c	201.5 ± 7.2 b–d	204.9 ± 6.6 ab	357.3 ± 7.4 a–f
M05	146.9 ± 2.8 a–c	265.8 ± 5.4 a	79.0 ± 2.2 ab	190.7 ± 3.2 a–c	19.8 ± 1.0 ab	84.7 ± 2.2 a–c	100.5 ± 2.6 a–c	205.2 ± 7.6 a–c	196.9 ± 2.7 ab	370.9 ± 9.7 a–c
M06	147.5 ± 1.9 a–c	252.8 ± 8.5 a–c	75.6 ± 1.9 ab	194.2 ± 5.2 a	18.2 ± 1.0 ab	81.8 ± 2.9 b–f	102.8 ± 2.5 ab	210.5 ± 7.6 ab	192.9 ± 4.7 a–c	369.4 ± 9.6 a–d
M07	149.2 ± 2.4 a–c	258.7 ± 5.7 ab	83.7 ± 1.9 a	188.7 ± 4.6 a–c	20.2 ± 1.2 a	84.3 ± 1.8 a–d	101.3 ± 3.8 a–c	206.3 ± 5.9 a–c	198.3 ± 5.3 ab	363.8 ± 9.2 a–e
M08	145.9 ± 3.9 a–c	232.6 ± 5.4 e	76.1 ± 2.1 ab	159.2 ± 2.8 fg	17.3 ± 1.0 ab	72.2 ± 2.7 gh	92.7 ± 2.6 bc	181.7 ± 5.2 ef	187.8 ± 6.5 bc	325.9 ± 7.8 hi
M09	150.4 ± 1.9 a–c	264.1 ± 6.2 ab	82.0 ± 1.9 ab	190.0 ± 4.7 a–c	19.0 ± 0.8 ab	86.0 ± 2.3 ab	98.6 ± 2.6 a–c	202.1 ± 7.4 b–d	206.8 ± 5.4 ab	378.7 ± 9.6 ab
M10	153.4 ± 2.9 ab	241.5 ± 4.8 c–e	83.5 ± 2.7 a	177.5 ± 5.7 c–e	20.6 ± 1.3 a	75.7 ± 2.2 f–h	105.3 ± 3.0 ab	190.6 ± 5.1 c–f	204.8 ± 7.0 ab	346.0 ± 9.1 d–h
M11	159.9 ± 2.9 ab	236.9 ± 3.9 de	85.7 ± 2.6 a	161.4 ± 3.3 fg	22.4 ± 1.5 a	72.5 ± 2.2 gh	111.4 ± 4.1 a	189.0 ± 6.7 c–f	216.0 ± 7.2 a	327.9 ± 6.2 g–i
M12	147.2 ± 3.5 a–c	242.1 ± 4.7 c–e	80.3 ± 2.6 ab	172.3 ± 3.4 d–f	19.4 ± 1.1 ab	79.2 ± 2.1 c–f	95.8 ± 3.1 a–c	201.5 ± 6.6 b–d	191.9 ± 6.7 a–c	338.7 ± 8.2 e–i
M13	151.1 ± 2.3 ab	252.5 ± 4.8 a–d	78.6 ± 1.7 ab	181.1 ± 5.1 a–e	20.8 ± 0.9 a	83.8 ± 2.6 a–e	97.8 ± 3.3 a–c	198.3 ± 3.1 b–e	202.3 ± 4.5 ab	360.9 ± 8.6 a–e
M14	156.3 ± 2.2 ab	261.0 ± 4.9 ab	81.6 ± 2.7 ab	187.9 ± 4.5 a–c	22.1 ± 1.3 a	86.5 ± 2.4 ab	98.2 ± 3.5 a–c	210.4 ± 5.7 ab	210.7 ± 6.3 ab	373.5 ± 6.7 ab
M15	149.6 ± 2.2 a–c	263.4 ± 6.7 ab	80.5 ± 2.2 ab	190.4 ± 6.2 a–c	19.4 ± 0.9 ab	88.1 ± 2.8 a	104.0 ± 2.7 ab	222.3 ± 6.5 a	204.4 ± 5.5 ab	381.2 ± 11.8 a
Region	791,546.0 ***		786,069.0 ***		294,879.0 ***		776,073.0 ***		1,901,564.0 ***	
Year	23.0 ns		71.7 ns		0.3 ns		132.4 ns		352.2 ns	
Region*year	49.2 ns		38.5 ns		9.1 ns		36.1 ns		121.2 ns	
Rep (region Year)	254.3 ns		124.8 ns		35.1 ns		129.3 ns		601.7 ns	
Genotype	726.3 **		805.0 **		159.4 **		850.9 ***		2747.5 ***	
Region*genotype	694.1**		714.3**		125.2**		549.1*		1902.5**	
Year*genotype	242.4 ns		112.1 ns		32.2 ns		219.3 ns		371.6 ns	
Region*year*genotype	215.1 ns		105.0 ns		34.9 ns		225.3 ns		619.8 ns	
Error	164.3		118.3		25.5		216.6		466.7	
CV (%)	6.4		8.4		10.1		9.9		7.8	
χ^2§^	0.13 ns		0.02 ns		0.05 ns		0.02 ns		0.02 ns	

For each trait, in each region, means with the same letter are not significantly different (slicing method, p < 0.05).

*ns* not significant.

*, **, and ***; significant at 0.05, 0.01, and 0.001 probability level, respectively; §; Bartlett’s test for homogeneity.

### Bakery quality

Protein content was significantly affected by region (salinity), genotype, and their interaction (Table 3). All genotypes had higher protein content in salinity stress conditions, although protein increment due to salinity stress was different in studied genotypes (Table 3). M07 and M12 showed the lowest (1.8%) and the highest (17.3%) protein increase, respectively. Zeleny sedimentation volume (%) showed a similar trend to protein content (Table 3), so that, it increased under salinity stress conditions in all genotypes except M06, C2, C3 (no change), and M07 (8% decrease). The bread volume (ml) was increased due to salinity stress in half of the genotypes, whereas it decreased in the other genotypes due to salinity stress (Table 3). The highest increase (24.9%) and the highest decrease (19.7%) of bread volume were observed in M1 and M09, respectively. Region and region × genotype had a significant impact on hardness index, so it increased due to salinity stress in all genotypes except C1 (2.2% decrease) and C3 (no change, Table 3). The result indicated that salinity stress increased wet gluten (%) in all genotypes. M10 and M08 showed the highest (47.8%) and the lowest (4%) wet gluten increment, respectively (Table 3) It was not observed a clear trend in the gluten index of studied genotypes in response to salinity stress. So that its change due to salinity stress ranged from a 76% decrease in M02 to a 67.4% increase in M03. The gluten elasticity of some genotypes was changed as affected by salinity stress (Table 3). It changed from normal to soft in M04, M06, M09, M10, M11, C2, and C3. While, it changed from hard to soft in M02, from normal to hard in M03, and from hard to normal in M08.

**Table 3:** Characteristics related to bakery quality of wheat genotypes (means and analysis of variance) in Karaj (no salinity stress) and Yazd (salinity stress).

Region	Genotype	Protein ± SE (%)	Zeleny sedimentation volume ± SE (%)	Bread volume ± SE (ml)	Hardness index ± SE	Wet gluten (%)	Gluten index ± SE	Gluten elasticity
Karaj	C1	11.4 ± 0.5 cd	21 ± 1 cd	511 ± 10.4 cd	45 ± 1.5 a	24 ± 2.1 a	42 ± 2.7 ij	N
	C2	11.5 ± 0.5 bc	25 ± 0.2 a	489 ± 10.4 de	43 ± 1.4 a–c	24 ± 2.1 a	50 ± 0.8 d–g	N
	C3	11.5 ± 0.5 bc	25 ± 0.2 a	487 ± 0.6 e	44 ± 0.1 ab	25 ± 0.3 a	52 ± 2.8 c–f	N
	M01	11.2 ± 0.6 e	21 ± 1 cd	442 ± 9.2 f	42 ± 1.6 a–c	24 ± 2.1 a	35 ± 1.2 k	N
	M02	11.3 ± 0.6 de	23 ± 1 a–c	510 ± 9.8 c–e	43 ± 1.4 a–c	21.86 ± a	75 ± 1.1 a	H
	M03	11.2 ± 0.6 e	22 ± 0.1 b–d	445 ± 10.4 f	42 ± 1.6 a–c	23 ± 2 a	43 ± 2.4 hi	N
	M04	11.5 ± 0.5 bc	22 ± 0.1 b–d	499 ± 5.8 c–e	43 ± 1.4 a–c	24 ± 2.1 a	57 ± 1.3 c	N
	M05	11.8 ± 0.4 a	22 ± 0.1 b–d	487 ± 0.6 e	41 ± 0.3 bc	24 ± 2.1 a	70 ± 2.3 ab	H
	M06	11.6 ± 0.5 b	24 ± 1 ab	580 ± 11 a	43 ± 1.4 a–c	24 ± 2.1 a	44 ± 0.1 g–i	N
	M07	11.4 ± 0.5 cd	25 ± 0.2 a	489 ± 10.4 de	41 ± 0.3 bc	24 ± 2.1 a	49 ± 2.8 e–h	N
	M08	11.2 ± 0.6 e	20 ± 1 d	500 ± 5.2 c–e	42 ± 1.6 a–c	25 ± 0.3 a	64 ± 2.7 b	H
	M09	11.2 ± 0.6 e	21 ± 1 cd	580 ± 11 a	43 ± 1.4 a–c	25 ± 0.3 a	54 ± 1.6 c–e	N
	M10	11.2 ± 0.6 e	21 ± 1 cd	499 ± 5.8 c–e	42 ± 1.6 a–c	23 ± 2 a	54 ± 1.6 c–e	N
	M11	11.4 ± 0.5 cd	21 ± 1 cd	541 ± 6.9 b	42 ± 1.6 a–c	24 ± 2.1 a	56 ± 1.5 cd	N
	M12	11 ± 0.6 f	22 ± 0.1 b–d	491 ± 9.2 de	40 ± 1.3 c	26 ± 1.8 a	54 ± 1.6 c–e	N
	M13	11.5 ± 0.5 bc	22 ± 0.1 b–d	518 ± 4 bc	42 ± 1.6 a–c	24 ± 2.1 a	46 ± 2.6 f–i	N
	M14	11.5 ± 0.5 bc	20 ± 1 d	502 ± 6.9 c–e	40 ± 1.3 c	25 ± 0.3 a	36 ± 2.9 jk	N
	M15	11.3 ± 0.6 de	20 ± 1 d	580 ± 11 a	42 ± 1.6 a–c	25 ± 0.3 a	52 ± 2.8 c–f	N
Yazd	C1	12.6 ± 0.1 ab	26 ± 0.9 bc	580 ± 11 a	44 ± 0.1 c	26 ± 1.8 e	55 ± 2.9 b	N
	C2	12.6 ± 0.1 ab	25 ± 0.2 cd	503 ± 4 ef	44 ± 0.1 c	29 ± 1.6 b–e	14 ± 2.9 f	S
	C3	12.6 ± 0.1 ab	25 ± 0.2 cd	500 ± 5.2 ef	44 ± 0.1 c	30 ± 2.3 a–e	16 ± 0.8 ef	S
	M01	12.8 ± 0.1 a	26 ± 0.9 bc	552 ± 9.2 bc	46 ± 1.6 a–c	32 ± 1.3 a–d	47 ± 0.3 cd	N
	M02	13 ± 0.2 a	29 ± 0.8 a	572 ± 2.9 ab	47 ± 0.2 ab	35 ± 1 a	18 ± 2.2 ef	S
	M03	12.5 ± 0.1 ab	26 ± 0.9 bc	489 ± 10.4 fg	47 ± 0.2 ab	31 ± 0.9 a–e	72 ± 0.8 a	H
	M04	12.6 ± 0.1 ab	25 ± 0.2 cd	572 ± 2.9 ab	44 ± 0.1 c	33 ± 2.3 a–c	22 ± 0.1 e	S
	M05	12.2 ± 0.2 bc	25 ± 0.2 cd	462 ± 2.3 h	48 ± 1.3 a	26 ± 1.8 e	76 ± 1.6 a	H
	M06	11.9 ± 0.3 cd	24 ± 1 cd	542 ± 11.5 cd	46 ± 1.6 a–c	30 ± 2.3 a–e	17 ± 2.8 ef	S
	M07	11.6 ± 0.5 d	23 ± 1 d	540 ± 4 cd	46 ± 1.6 a–c	26 ± 1.8 e	51 ± 2 bc	N
	M08	11.7 ± 0.5 cd	25 ± 0.2 cd	560 ± 8.1 a–c	46 ± 1.6 a–c	26 ± 1.8 e	49 ± 2.8 b–d	N
	M09	12.6 ± 0.1 ab	26 ± 0.9 bc	466 ± 9.8 gh	45 ± 1.5 bc	33 ± 2.3 a–c	21 ± 2.4 e	S
	M10	12.5 ± 0.1 ab	25 ± 0.2 cd	466 ± 9.8 gh	45 ± 1.5 bc	34 ± 1.2 ab	14 ± 2.9 f	S
	M11	12.7 ± 0.1 ab	26 ± 0.9 bc	540 ± 4 cd	46 ± 1.6 a–c	33 ± 2.3 a–c	20 ± 2.7 ef	S
	M12	12.9 ± 0.2 a	28 ± 0.3 ab	450 ± 8.1 h	45 ± 1.5 bc	31 ± 0.9 a–e	44 ± 0.1 d	N
	M13	12.6 ± 0.1 ab	26 ± 0.9 bc	504 ± 11 ef	46 ± 1.6 a–c	30 ± 2.3 a–e	52 ± 2.8 bc	N
	M14	12.5 ± 0.1 ab	24 ± 1 cd	501 ± 11.5 ef	44 ± 0.1 c	28 ± 0.9 c–e	50 ± 0.8 b–d	N
	M15	12.5 ± 0.1 ab	25 ± 0.2 cd	522 ± 5.8 de	45 ± 1.5 bc	27 ± 2.2 d–e	48 ± 2.2 cd	N
Region		32.3***	320.3***	243.8**	280.3***	850.1***	508.1***	
Genotype		0.2***	6***	516.4***	3.2 ns	14.7*	686.7***	
Region*genotype		0.3***	8.3***	479.9***	5.9*	14.3*	896.3***	
CV (%)		2.1	5.1	2.8	4	10.1	8.2	

For each trait, in each region, means with the same letter are not significantly different (slicing method, p < 0.05).

*H* hard, *N* normal, *S* soft.

### Grain yield

**Figure 1:**
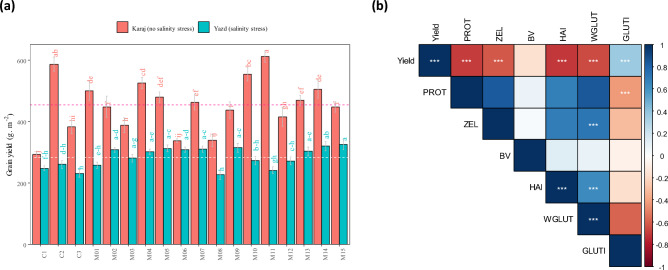
(**a**) Grain yield of studied wheat genotypes in Karaj (without salinity stress) and Yazd (salinity stress) mean data of 2018, 2019, and 2020 years. Red dashed-line; mean grain yield in Karaj (no salinity stress), white dashed-line; mean grain yield in Yazd (salinity stress). Means with the same letter are not significantly different (Slicing method, p < 0.05). (**b**) Graphic view of pearson correlation matrix between grain yield and and bakery quality characteristics of wheat genotypes (correlation plot was was generated using corrplot package v. 0.92 in R v. 4.2.2). *PROT* protein content, *ZEL* Zeleny sedimentation, *BV* bread volume, *HAI* hardness index, *WGLUT* wet gluten, *GLUTI* gluten index. *, **, and *** significant at p < 0.05, p < 0.01, and p < 0.001, respectively.

The results indicated that the region, genotype, and their interaction had a significant effect on grain yield (Table 4). Grain yield of all genotypes was lower under salinity stress conditions (Yazd) though the reduction due to salinity was not the same in all genotypes (Fig. 1a). M06 and M11 mutants showed the lowest (6.1%) and the highest (60.7%) decrement of grain yield, respectively. Indeed, M11 had the highest grain yield under no salinity stress conditions and the highest grain yield reduction due to salinity stress. As given in Fig. 1a, genotypes are classified into four groups based on comparison of each genotype grain yield in each salinity conditions to average of grain yield of all genotypes in the same salinity conditions; (1) genotypes with high (more than average of all genotypes) grain yield under salinity stress conditions but low grain yield under no salinity stress including M02, M06, M09, and M15; (2) genotypes with high grain yield in both conditions (genotypes with high grain yield stability) including M04, M05, M07, M13, and M14, (3) genotypes with high grain yield under no salinity stress conditions but low grain yield under salinity stress includingC1, M1, M10, and M11, and (4) genotypes with low grain yield in both conditions including C1, C3, M03, M08, and M12.

**Table 4:** Analysis of variance for grain yield of studied wheat genotypes in Karaj (no salinity stress) and Yazd (salinity stress).

Source	DF	MS	
Region	1	2,375,195.0	***
Year	2	1223.9	ns
Region*year	2	155.0	ns
Rep (region year)	12	2087.4	ns
Genotype	17	42,203.0	***
Region*genotype	17	36,439.0	***
Year*genotype	34	4022.8	ns
Region*year*genotype	34	4389.5	ns
Error	204	3943.2	
CV (%)		17.1	
χ^2§^		0.29	ns

*ns* not significant.

***Significant at 0.001 probability level.

^§^Bartlett’s test for homogeneity.

**Figure 2:**
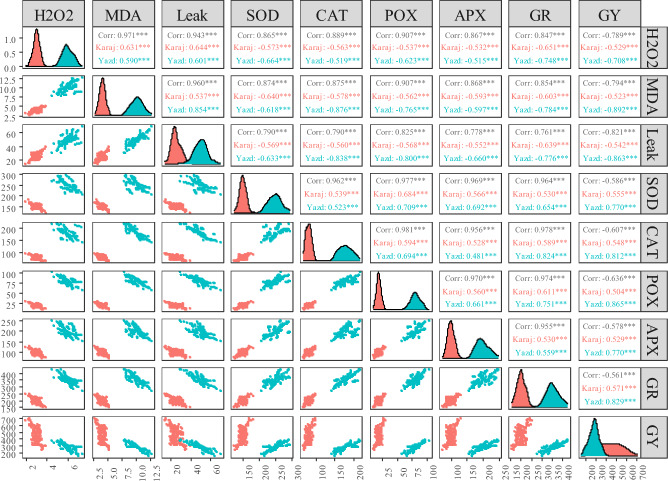
Graphic view of pearson correlation matrix between grain yield and and bbiochemical traits of wheat genotypes. *MDA* malondialdehyde, *leak* ion leakage, *SOD* superoxid dismutase, *CAT* catalase, *POX* peroxidase, *APX* ascorbate peroxidase, *GR* glutathione reductase, *GY* grain yield. *, **, and *** significant at p < 0.05, p < 0.01, and p < 0.001, respectively (correlation matrix was generated using ggally package v. 2.1.2 in R v. 4.2.2).

Results also showed a negative and significant correlation between grain yield with protein content, Zeleny sedimentation volume, hardness index, and wet gluten, while the correlation between grain yield and gluten index was positive and significant (Fig. 1b). As given in Fig. 2 grain yield was positively correlated to antioxidant activity and negatively was associated to ion leakage and content of H_2_O_2_ and MDA although correlation was greater in salinity stress conditions. In addition, it was observed that there was a positive and significant correlation between ion leakage and content of H_2_O_2_ and MDA also there was a positive and significant correlation among enzymatic antioxidants.

**Figure 3:**
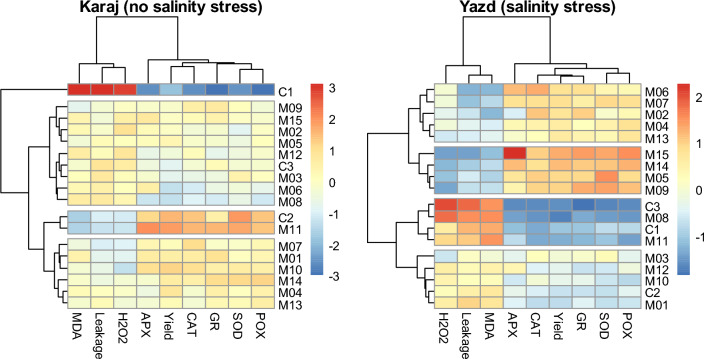
Heat map view of studied wheat genotypes based on biochemical traits and grain yield data in no salinity stress (Karaj) and salinity stress (Yazd) conditions (heat map was generated using pheatmap package v. 1.0.12 in R v. 4.2.2).

Clustering the genotypes based on biochemical traits and grain yield in each salinity conditions revealed that genotypes were classified into four groups (Fig. 3). M05, M09, M14, and M15 had the highest grain yield (318 g m^-2^ on average) and the most antioxidant activity in salinity stress conditions. In no salinity stress conditions, also the maximum grain yield was belonged to C2 and M11 (599 g m^-2^ on average).

## Discussion

In the current study lipid peroxidation (MDA accumulation), H_2_O_2_ content and ion leakage were increased due to salinity stress however, their increment were not the same in all genotypes. Salinity stress induces the production of reactive oxygen species (ROS) such as superoxide radical (${\mathrm{O}}_{2}^{\cdot -}$), hydrogen peroxide (H_2_O_2_) and hydroxyl radical (${\mathrm{OH}}^{\cdot }$) in the plant; ROS are responsible for the peroxidation of membrane lipids and as a result membrane decay and increased ion leakage. In addition, ROS damage other essential macromolecules, photosynthetic pigments, protein, DNA and lipids^23,24^. It has been reported that salt-tolerant genotypes produce smaller amount of ROS rather than salt-sensitive ones^17^ or they have a more efficient antioxidant systems^25^. In the current study M02, M06, and M15 had the lowest ion leakage, MDA and H_2_O_2_ content increment due to salinity stress and they also a grain yield greater than average grain yield of all genotypes under salinity stress condition nevertheless their grain yield was low in no salinity stress conditions. Therefore, these mutants are suitable for salinity stress conditions or they can be used in plant breeding programs to increase tolerance to salt stress.

Our finding showed that activity of different enzymatic antioxidants including SOD, CAT, APX, POX, and GR was increased by salinity stress in all genotypes however, M15, M09, M06, and M05 had a greater antioxidant activity enhancement. In the aerobic metabolism of different plant organelles, including chloroplasts, mitochondria, peroxisomes, plasma membrane and cell wall, reactive oxygen species are produced as byproducts.^26–29^. Although ROS play a signaling role in low concentrations when their amount rises due to environmental stress, the plant must scavenge them to reduce oxidative damage to different organelles^29,30^. It has been well documented that different abiotic stresses such as drought and salinity induce oxidative stress in different crops. Plants cope with oxidative stress through triggering antioxidant systems including enzymatic and nonenzymatic antioxidants^31,32^. In addition, our findings showed that POX, CAT, and CAT had the highest increase in activity due to salinity stress, respectively. Several studies have proven that increasing the activity of antioxidant enzymes play an essential role in elevating tolerance to salt stress^33–35^.

The salinity stress increased the protein content of all genotypes; M07 and M12 showed the lowest (1.8%) and the highest (17.3%) protein enhancement, respectively. Our results are in accordance with Houshmand et al., (2005) who reported that salinity stress increased grain protein of wheat genotypes. It has been reported that salinity stress limits the leaf area index and the ability of the plant to remove dry matter during the grain filling period leading to less starch growing in the grain and then higher protein concentration^37^. Zeleny sedimentation volume (%) showed almost a similar trend to protein content, so that, it increased under salinity stress conditions in all genotypes except M06, control2, control3 (no change), and M07 (8% decrease). Hardness index increased due to salinity stress in all genotypes except control 1 (2.2% decrease) and control 3 (no change). The amount of Zeleny sediment volume describes the degree of sedimentation of the suspended flour in the lactic acid solution over a standard period of time, and this is considered a measure of the quality of the baking. The rate of sedimentation of the flour suspension is affected by the swelling of the gluten part of the flour in the lactic acid solution. Both higher gluten content and better gluten quality result in slower sedimentation and higher values of the Zeleny test. The sedimentation value of flour depends on the protein composition of wheat and is mainly related to the protein content, the hardness of the wheat, and the volume of the pan and hearth loaves^38^. The salinity stress increased wet gluten percentage in all genotypes. M10 and M08 showed the highest (47.8%) and the lowest (4%) wet gluten increment, respectively. Similar to our results, the gluten content of wheat genotypes increased by salinity stress^36^.

**Figure 4:**
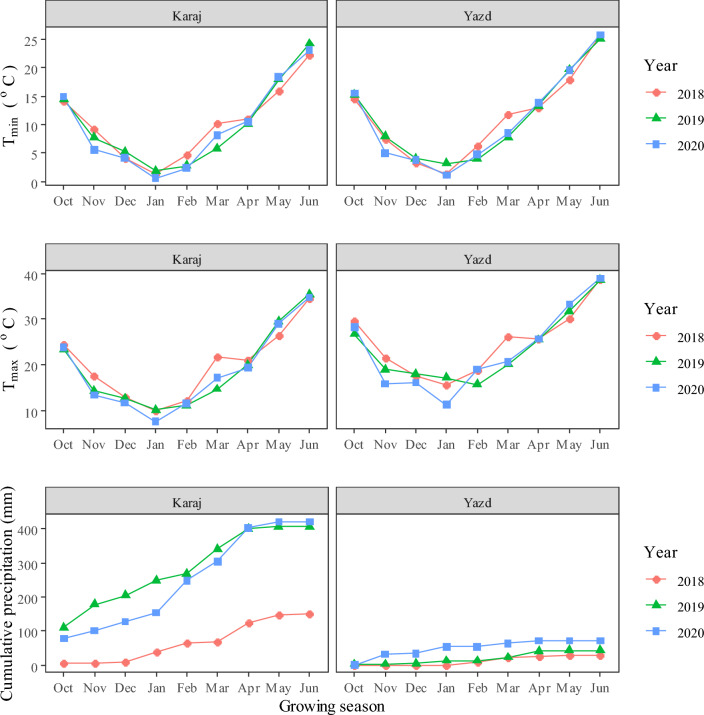
Meteorological data of studied regions during three growing seasons in Karaj (no salinity stress) and Yazd (salinity stress).

Our results showed that the grain yield of all genotypes was significantly higher in no salinity stress conditions. These results are similar to previous studies on the effect of salinity stress on wheat^4,19,37,39,40^. Under salinity stress, high osmotic stress, disruption of nutrient uptake, and ion toxicity cause to reduce cell turgor pressure, limit growth, and decrease grain yield of wheat^41,42^. However, not all of the differences between the two regions were associated with salinity stress. Weather conditions also affected the grain yield of genotypes. As shown in Fig. 4, cumulative precipitation was remarkably higher in Karaj in all growing seasons. Although in the current study, water requirement was met by irrigation, more precipitation certainly has a positive effect on grain yield. The mean temperature during the growing season was 14.7 °C in Karaj and 17.5 °C in Yazd. Also, there were 49 days with a temperature greater than 35 °C in Karaj (about 15 days per growing season), while the number of days with a temperature greater than 35 °C were 95 days (about 31 days per growing seasons). Day numbers with temperatures lower than 0 °C were almost similar for two regions (26 days in Karaj and 29 days in Yazd). This information indicated that in addition to salinity and precipitation, temperature also was more favorable in Karaj.

Mutants showed a different response to salinity stress than controls, so some of them had higher grain yield than control cultivars under salinity stress conditions while the others had an equal to or a lower grain yield than control cultivars. Genetic diversity is an essential prerequisite for developing salt-tolerant wheat genotypes^43^. However, the genetic base of salt-tolerant wheat breeding is narrow, and it limits the progress of salt tolerance in wheat^19^. As shown in the current study, increasing genetic diversity using mutation breeding with gamma irradiation can help to improve salt tolerance in wheat. Likewise, other researchers have used gamma irradiation to increase the genetic diversity of wheat to tolerate salinity stress^44^.

## Conclusion

The results of the current study revealed that salinity stress elevated antioxidant activity and decreased grain yield, contrary to baker quality that promoted by salinity stress. In addition, it was found that M05, M09, M14, and M15 had the highest grain yield and the most antioxidant activity in salinity stress. Therefore, these mutants have the potential to be introduced as a new salt-tolerant variety after additional tests in saline areas.

## Materials and methods

### Mutants

In order to evaluate wheat mutants under control conditions (without salinity stress) and salinity stress, this experiment was performed in the form of randomized complete blocks with three replications during 2018, 2019, and 2020 growing seasons. For this experiment, 15 wheat mutants and 3 control cultivars (Arg; C1, Bam; C2, and Narin; C3) were used (Table 5). Control cultivars are originated from temperate and warm regions, and they are relatively salt tolerant^45^. To produce wheat mutants, mutations were made using gamma irradiation with doses of 150 and 200 Gy on Arg and Bam cultivars (Table 6). After that, for several consecutive generations, the mutants were cultivated, and selection was made among them based on their morphological traits and grain yield. Finally, the top 15 mutants were selected for the current study. The fifth to seventh generations (M05 to M07 generations mutants) were planted for this study in 2018, 2019, and 2020 growing seasons, respectively.

**Table 5:** Wheat mutants, their maternal cultivar, and gamma irradiation doses for inducing mutation.

Mutant	Maternal cultivar/gamma dose	Mutant	Maternal cultivar/gamma dose	Mutant	Maternal cultivar/gamma dose
M01	Bam/150 Gy	M06	Arg/150 Gy	M11	Arg/200 Gy
M02	Arg/200 Gy	M07	Arg/200 Gy	M12	Bam/200 Gy
M03	Bam/200 Gy	M08	Bam/200 Gy	M13	Arg/150 Gy
M04	Arg/200 Gy	M09	Arg/200 Gy	M14	Arg/200 Gy
M05	Arg/150 Gy	M10	Arg/200 Gy	M15	Arg/150 Gy

**Table 6:** Control cultivars and their pedigrees.

Cultivar	Pedigree
Arg	Inia/22–66-1
Bam	Vee “s”/Nac//1-66-22/Vee “s”/Nac* T.Aest/5/Ti/4/La/3/Fr//Kal/Gb
Narin	Alvd//Aldan/Ias58/22/3–66-1

### Regions

**Figure 5:**
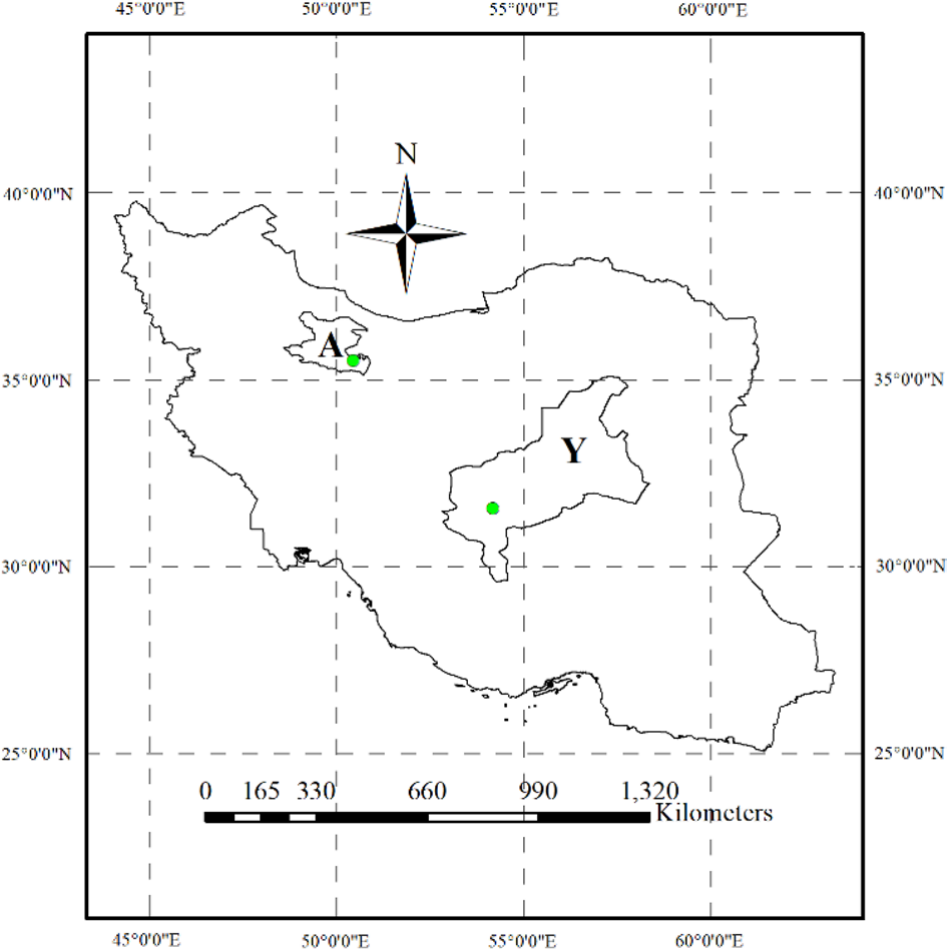
Location of the study area, A; Alborz (Karaj), Y; Yazd (Ardakan). The points on the map show the coordinates of the experimental farms (Map was generated using ArcMap, ArcGIS, v. 10.6).

To study the genotypes under control conditions (without salinity stress), genotypes were cultivated in the research farm of the Agricultural, Medical and Industrial Research Institute, Karaj, Alborz, Iran (35°49′ N, 50°44′ E). To study the genotypes under salinity stress conditions, they were cultivated in the research field of the seed and plant improvement research department, Yazd Agricultural and Natural Resources and Education Center, Ardakan, Yazd, Iran (31°54′ N, 54°16′ E). Location of the studied areas are shown in Fig. 5.

### Infield practices

Before performing the experiment in both regions, a soil sample was prepared from a depth of 0–30 cm, and the physicochemical properties of the soil were measured. The electrical conductivity of irrigation water and soil properties of the both regions during three years of study were given in Table 7. Meteorological data of studied regions during three growing seasons was also given in Fig. 4.

**Table 7:** Soil and irrigation water properties.

Region	Year	Irrigation water EC (dS m^−1^)	Soil EC (dS m^−1^)	pH	N (%)	P (mg kg^−1^)	K (mg kg^−1^)
Karaj	2018	1.4	2.4	7.2	0.07	10.5	95.8
	2019	1.6	2.7	7.5	0.09	12.3	103.2
	2020	1.8	3	7.7	0.07	13.4	110.7
Yazd	2018	5.9	9.9	8.3	0.1	17.6	87.8
	2019	6.3	10.1	8.3	0.09	18.8	90.6
	2020	6.5	10.1	8.5	0.08	18.5	97.3

Planting was done manually with a density of 450 plants per square meter in plots with six square meters area (1.2 m × 5 m) in the late October of each year. Irrigation was done as surface irrigation and according to the region’s custom in five stages during the growing season. The required amount of fertilizers in both regions was provided to the plant, based on the soil test results (150 kg ha^−1^ N, 100 kg ha^−1^ P, 100 kg ha^−1^ K). One-third of nitrogen fertilizer and all phosphorus and potassium fertilizers were given to the plant before planting and the rest of nitrogen in two stages after emergence and stem elongation. Weeding weeds was done manually at tillering and stem elongation stages. No disease or pest was observed in the research fields during the study.

### Biochemical traits

At the anthesis stage, for biochemical measurements, ten random selected flag leaves were taken in each plot. For this purpose H_2_O_2_, MDA, SOD, CAT, POX, APX, and GR was assayed using methods described by Mukherjee and Choudhari (1983)^46^, Rao and Sresty (2002)^47^, Beuchamp and Fridovich (1971)^48^, Bergmeyer (1962)^49^, Herzog and Fahimi (1973)^50^, Nakano and Asada (1981)^51^, Foyer and Halliwell (1976)^52^, respectively.

### Ion leakage

For measuring ion leakage, at the anthesis stage, ten one-cm^2^-piece was taken from flag leaves in each plot and were immersed in distilled water for 20 min at the room temperature. Samples were washed thoroughly then placed in 20 mL of fresh distilled water for 1 h and then the initial electrical conductivity (EC1) was measured. To measure EC2, the samples were boiled for 5 min, cooled to room temperature and the conductivity was measured again. Ion leakage (IL) was calculated as IL = (EC1/EC2) × 100^53^.

### Grain yield

At the time of physiological maturity, after removing the marginal plants, the plants were harvested from three square meters in the center of each plot, and after drying in the open air for a week to equalize the moisture of the samples, threshing was done, and grain yield was measured.

### Bakery quality features

Traits related to bakery quality, including protein percentage, Zeleny sediment volume, bread volume, hardness index, wet gluten, gluten elasticity, and gluten index, were measured in the grain chemistry laboratory of Seed and Plant Research Improvement Institute, Karaj, Iran, following the standards of International Association for Cereal Chemistry (ICC).

### Statistical design and data analysis

The data homogeneity among different years was evaluated using the Bartlett test. Shapiro–Wilk test was used to evaluate the normality distribution of data. Data were analyzed using the GLM procedure in the SAS environment (SAS 9.4). To do this genotype, and region were considered as fixed factors, and year was considered as a random factor. The least significant difference (LSD) was also used for mean comparison. pheatmap (v. 1.0.12)^54^, ggally (v. 2.1.2)^55^, and corrplot (v. 0.92)^56^ packages also were used to draw Genotype*Trait, correlation matrix and correlation plot, respectively, in the R (v. 4.2.2) programming environment.

### Ethical approval

We confirm that all the experimental research and field studies on plants (either cultivated or wild), including the collection of plant material, complied with relevant institutional, national, and international guidelines and legislation. All of the material is owned by the authors and/or no permissions are required.

## Data Availability

The datasets used and/or analyzed during the current study available from the corresponding author on reasonable request.

## Abbreviations

APX: Ascorbate peroxidase
BV: Bread volume
CAT: Catalase
GLUTI: Gluten index
GR: Gluthation reductase
H_2_O_2_: Hydrogen peroxide
HAI: Hardness index
Leak: Ion leakage
MDA: Malondialdehyde
POX: Peroxidase
PROT: Protein content
SOD: Superoxide dismutase
WGLUT: Wet gluten
ZEL: Zeleny sedimentation
